# Categorization of household drinking water and sanitation service levels and associated determinants in Uganda

**DOI:** 10.1038/s41598-026-37203-9

**Published:** 2026-01-23

**Authors:** Betty Nakibuule, Henry Musoke Semakula, Denis Nseka, Hannington Wasswa, Carol Aboda, Precious Nampereza, Lydia Nabawanuka Namakula, Rawlance Ndejjo

**Affiliations:** 1 Department of Geography, Geo-informatics and Climatic Sciences, Makerere University, P. O Box 7062, Kampala, Uganda; 2 Department of Disease control and Environmental Health, Makerere University, P. O Box 7072, Kampala, Uganda

**Keywords:** JMP service ladders, SDG 6, Water and sanitation services, WASH determinants, Uganda, Risk factors, Sustainability

## Abstract

Achieving universal access to safe drinking water and sanitation remains a major challenge in Uganda, where persistent socioeconomic and spatial inequalities limit progress toward Sustainable Development Goal 6. While previous studies have largely focused on localized settings, national-level evidence applying the WHO/UNICEF Joint Monitoring Programme (JMP) service ladder framework is limited. This study aimed to address this gap. This study used secondary data from the 2018–2019 Uganda Malaria Indicator Survey. A total of 8,925 households were included, with data drawn from the Household Recode file. The dependent variables were household sources of drinking water and types of toilet facilities and these were classified according to the WHO/UNICEF JMP service ladder framework into safely managed, basic, limited, unimproved, and no service categories. Independent variables comprised a range of individual and household-level characteristics. Descriptive statistics were used to summarize key variables, while chi-square tests examined bivariate associations between the dependent and independent variables. Multivariable ordinal logistic regression was then applied to estimate the adjusted effects of the determinants on water and sanitation service levels. All analyses accounted for the complex survey design and sampling weights and were conducted using Stata version 18. Most Ugandan households relied on basic drinking water services (67.8%), while safely managed water remained low (8.8%) and was almost absent in regions such as Karamoja. Sanitation access was dominated by limited services (49.7%), with only 43.6% achieving safely managed sanitation. The significant determinants associated with JMP service levels were identified. Higher education, household wealth, male headship, and residence in central and western regions strongly increased the likelihood of accessing higher service levels. Rural areas and refugee settlements consistently exhibited the lowest service levels, reflecting structural deficits in infrastructure, affordability, and service reliability. This study shows that safely managed drinking water and sanitation remain limited in Uganda, with persistent inequalities driven by education, wealth, gender, and region. Targeted investments and pro-poor, equity-focused policies are urgently needed to improve service levels in underserved rural areas and refugee settlements. Strengthening infrastructure, reducing affordability barriers, and supporting context-specific water and sanitation programming will be essential for accelerating progress toward universal and safely managed services.

## Introduction

 Access to safe drinking water and adequate sanitation facilities is universally recognized as a foundational component for preventing diseases, promoting good health and wellbeing, enhancing education, boosting economic productivity, ensuring dignity, safety, and environmental sustainability^1^. These benefits highlight the critical need for continued investment and policy focus on enhancing water and sanitation infrastructure worldwide^2^. Despite these benefits, a significant portion of the global population still lacks access to clean water and adequate sanitation. As of 2022, over 2.2 billion people lacked safely managed drinking water, 703 million had no basic water services, 3.5 billion lacked safely managed sanitation, and 1.5 billion had no basic sanitation services^3^. Additionally, more than 2 billion people globally, live in water-stressed regions, a challenge exacerbated by climate change and population growth^4^. These disparities hinder progress toward achieving the Sustainable Development Goals (SDGs), particularly SDG 6, which aims to ensure availability and sustainable management of water and sanitation for all by 2030^5^.

The crisis is particularly more acute in Africa, where water and sanitation challenges remain widespread. More than 411 million people lack basic drinking water services, 208 million practice open defecation, and 829 million lack basic hygiene services, with nearly three-quarters of the population lacking access to safely managed sanitation^6^. Over 296 million people rely on unprotected wells and springs, and 115 million draw untreated surface water from lakes, rivers, and streams often contaminated due to open defecation and inadequate waste management^7^. Sub-Saharan Africa (SSA) bears a significant burden, with an estimated 7.8% of deaths due to diarrheal diseases linked to unsafe water, sanitation, and hygiene (WASH), with a risk factor attribution of 95.9%^7^. Furthermore, intermittent water supply is a persistent issue, with many households accessing water for only a few hours per day^8^.

Uganda exemplifies these regional challenges, with 18.9% of households depending on unimproved water sources and 49.7% lacking access to safely managed services^9^. These deficiencies expose communities to severe public health risks, increasing the prevalence of waterborne diseases such as cholera, dysentery, typhoid, hepatitis A, diarrheal diseases, and parasitic infections^10,11^. Beyond health implications, inadequate water and sanitation services contribute to economic losses, with global estimates indicating an annual loss of $260 billion due to these deficiencies^12^ Socially, the crisis exacerbates gender inequalities, and leads to environmental challenges such as water pollution^13^. The education sector is also affected, as students’ health, school attendance, and academic performance suffer^14^.

In SSA, numerous studies have documented persistent challenges in achieving universal access to safe water and adequate sanitation. Two recent review studies showed that WASH challenges in SSA stem from intertwined institutional weaknesses such as poor policy coordination, weak regulatory enforcement, and limited community participation coupled with economic and political constraints, including chronic underinvestment, corruption, and inequitable cost-sharing arrangements^15,16^. These structural limitations are further compounded by geographical barriers in informal settlements and remote rural areas, where extending water and sanitation infrastructure remains both costly and technically demanding, resulting in persistent service gaps^15,16^. Evidence from another systematic review focusing on internally displaced persons (IDP) camps across SSA reported additional barriers, including insecurity in conflict-affected zones, inadequate community involvement in Water, Sanitation and Hygienic planning, significant logistical challenges in hard-to-reach areas, and an overreliance on donor-driven, short-term interventions that undermine long-term sustainability^17^. Regional SDG 6 assessments further reveal slow progress between 2015 and 2020, only minimal improvements were observed, with more than half of SSA’s population still lacking access to safely managed drinking water, improved sanitation facilities, basic hygiene services, and safe wastewater treatment^18^. This regional assessment attributed these deficits to inadequate and aging infrastructure, weak governance and regulatory systems, rapid population growth, and chronic underinvestment in the WASH sector. Consequently, large segments of the population remain dependent on unreliable, unsafe, or informal water and sanitation services, perpetuating health risks and deepening socio-economic inequalities^19^.

In Uganda, a growing body of research on water and sanitation has been conducted. Nationally, one study developed a WASH Resource Index (WRI), mapped high-risk WASH practices and child diarrhea geographically, and assessed how the WRI relates to child diarrhea at both cluster and individual levels^20^. Another study focused on identifying the key determinants influencing household transitions across different levels of the Drinking Water Supply Systems (DWSS) service ladder^21^. Other studies have focused particularly within informal urban settlements. For example, Ssemugabo et al.^22^ documented pronounced inequities in Kampala’s slums, showing that the quality and reliability of water services remain substantially below national averages, despite the settlements’ physical proximity to urban infrastructure. Additional evidence from a slum-based study in Kampala highlighted that individual-level factors, including knowledge, beliefs, language barriers, and financial limitations hinder adoption of WASH interventions^23^. The same study revealed broader social and structural constraints such as negative peer influences, unsupportive household and community environments, cultural norms, limited space for sanitation facilities, and political interference, all of which collectively reduce the uptake of WASH services^23^. Similarly, Dickson-Gomez et al.^24^ found that even where facilities are physically present, they often fail to provide safe, continuous, and dignified services due to operational breakdowns, overcrowding, poor maintenance, and environmental contamination. A more recent and comprehensive investigation applied the Joint Monitoring Programme (JMP) service ladder classification in the Kinawataka informal settlement and reported that drinking water services were predominantly basic (75%), while sanitation services were largely limited (88%), with only 12% and 7% meeting safely managed standards, respectively^25^. The same study underscored that the existence of water points or latrines alone does not ensure safely managed service levels; rather, factors such as reliability, distance, cost, and the number of users per facility critically shape the quality and safety of WASH access^25^.

Although substantial research on water and sanitation in Uganda has examined WASH inequities, high-risk practices, health outcomes, and service conditions, most studies report only the type or functionality of water and sanitation technologies (e.g., pit latrines, boreholes, tap water) rather than categorizing these services according to the WHO/UNICEF JMP service ladder, namely safely managed, basic, limited, unimproved, or surface water^26–28^. Moreover, much of the current evidence is drawn from Kampala’s informal settlements or small community-based studies, and while these provide valuable insights, they do not offer a nationally representative assessment of household drinking water and sanitation service levels across Uganda. Moreover, previous studies have not comprehensively applied the WHO/UNICEF Joint Monitoring Programme (JMP) service ladder to categorize service levels at the national scale, nor have they systematically modelled the demographic and socio-economic determinants driving variations in these service levels. Importantly, no study to date has utilized the nationally representative 2018 Malaria Indicator Survey (MIS), which uniquely captures both general households and refugee settlements, to evaluate water and sanitation access using JMP standards. As a result, critical national-level patterns, regional disparities, and vulnerabilities among refugee populations remain underexplored.

This study addresses these critical gaps by providing the first national-level JMP-based categorization and statistical modelling of drinking water and sanitation service levels in Uganda. Using a nationally representative dataset (i.e. 2018–2019 Malaria Indicator survey dataset), the study aimed to: (1) categorize household drinking water and sanitation service levels in Uganda using the WHO/UNICEF Joint Monitoring Programme (JMP) framework^29^; and (2) model the demographic and socio-economic determinants influencing these service levels across the country. This study makes three key important and novel contributions to the WASH literature in Uganda and sub-Saharan Africa. First, this study is the first to categorize drinking water and sanitation service levels across all Ugandan households using the internationally standardized WHO/UNICEF JMP framework. This enables direct alignment with SDG 6 monitoring and global comparability. Second, by including refugee households captured in the MIS 2018, the study provides unique insights into WASH inequalities across both host communities and refugee populations, an area rarely examined at national scale. Third, the study’s results provide actionable information for national planners, development partners, and humanitarian actors, supporting more efficient resource allocation and targeted strategies to reduce service inequities.

## Materials and methods

### Study area

**Fig. 1:**
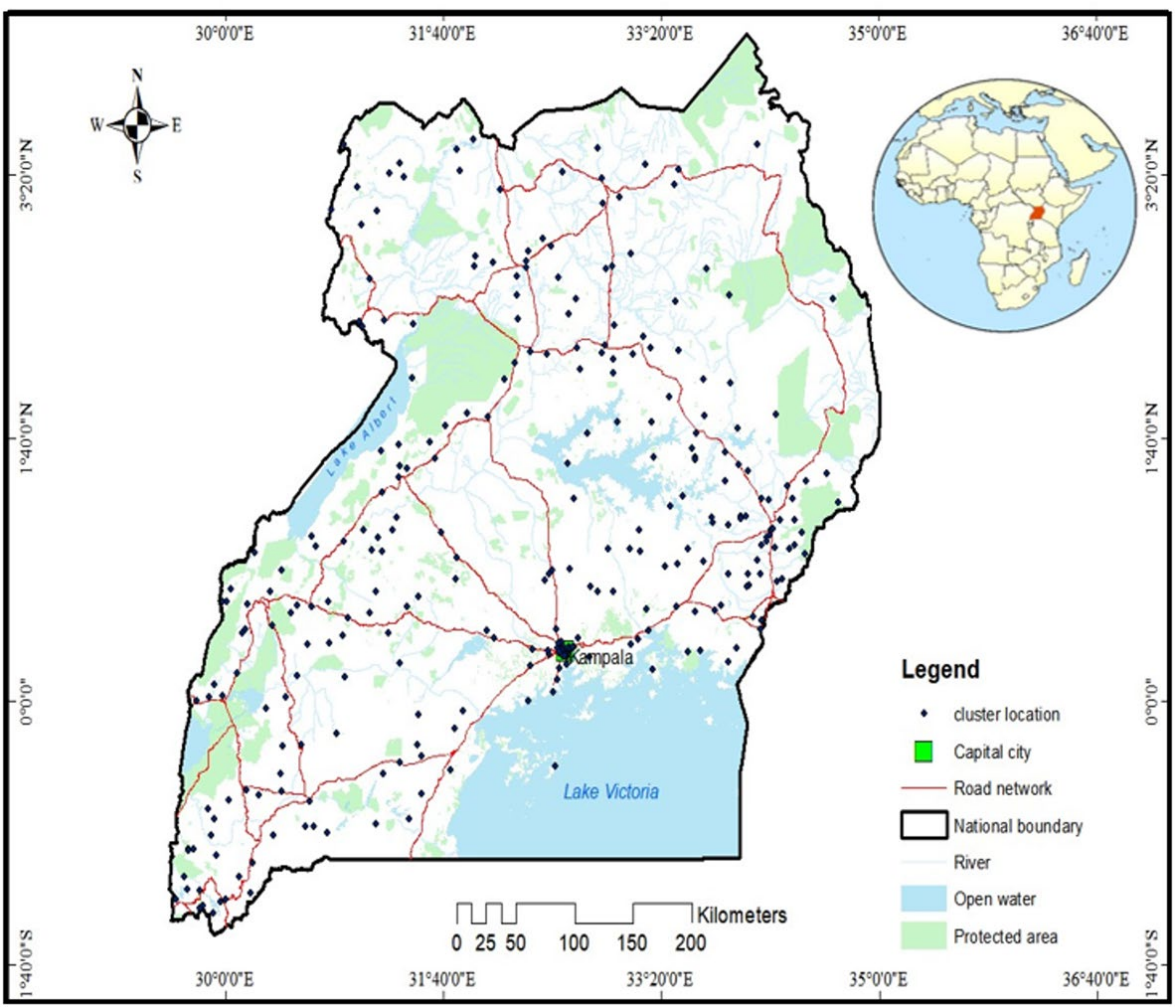
Study area and UMIS cluster locations. The map was created using UMIS cluster GPS location data and generated in ArcGIS version 10.8 (Esri, Redlands, CA, USA; https://www.esri.com). DHS cluster coordinates are geographically displaced to protect respondent confidentiality.

The study focused on Uganda, a landlocked country situated in Eastern Africa^30^. Uganda shares borders with Kenya to the east, South Sudan to the north, the Democratic Republic of Congo to the west, Rwanda to the southwest, and Tanzania to the south^31^. The country spans a total area of 241,551 square kilometers, including 200,523 square kilometers of land^31^. Uganda is home to over 1.5 million refugees from countries such as South Sudan, Somalia, Sudan, the Democratic Republic of Congo, Ethiopia, Eritrea, Rwanda, and Burundi^32^. With a projected population of 44.2 million people^33^, Uganda is divided into 15 regions as depicted in Fig. 1.

### Data sources

This study analyzed secondary data from the 2018–2019 Uganda Malaria Indicator Survey (UMIS) which adopted a cross sectional survey design. The Malaria Indicator survey dataset was obtained from the Demographic and Health Survey program (DHS) after permission was granted. This DHS program is responsible for collecting and disseminating accurate, nationally representative data on health and population in developing countries^34^. The UMIS dataset is the first national wide survey in Uganda to include households in refugee settlements^34^. Data was extracted from the household record file and the unit of analysis was a household. Standardized questionnaires were designed to collect the demographic, social, economic and environmental information of the surveyed households^35^.

### Study population and sample size

A total of 8957 households participated in the Uganda Malaria Indicator Survey (UMIS), conducted between December 2018 and February 2019. The survey utilized the 2014 Uganda Population and Housing Census frame and employed a two-stage stratified sampling method. In the first stage, the sampling frame was stratified by geographic areas (urban, rural, and refugee settings) based on the enumeration areas from the 2014 census. This stage involved selecting clusters from each stratum and listing households within the chosen clusters. In the second stage, households to be interviewed were selected from these listings. This study utilized the Household Recode (HR file) dataset, which was obtained from the DHS Program website (https://dhsprogram.com/). The Household Recode dataset contained both male and female-headed households that had complete data on access to drinking water sources and sanitation facilities. Households were eligible for this study if they participated in the 2018–2019 Uganda Malaria Indicator Survey and provided reliable responses to key WASH indicators. Households with incomplete or missing data on water and sanitation variables were excluded. Additionally, those that did not meet the survey’s eligibility criteria or had inconsistencies in reporting were omitted from the analysis. After applying the inclusion and exclusion criteria, the final sample size for this study comprised 8925 households.

### Variables

#### Dependent variables

This study utilized two dependent variables: sources of drinking water and types of toilet facilities. In line with World Health Organization and United Nations Children’s Fund (WHO/UNICEF) Joint Monitoring Programme (JMP) standards, the study classified household drinking water sources and toilet facility types into internationally recognized service ladders (Table 1). These classifications were derived from the WHO/UNICEF Progress Reports on Household Drinking Water, Sanitation, and Hygiene (2000–2024)^29,36^, which provide the latest global benchmarks for tracking SDG 6. This standardized classification ensured comparability with global monitoring systems and provided a robust framework for assessing service inequality across Uganda. It also enabled a more nuanced interpretation of access beyond simple technology type, capturing important aspects such as reliability, safety, and the burden of collection time.

**Table 1:** Categorization of dependent variables based on the drinking water and sanitation service levels of the JMP framework.

Service levels	Description
a) **Drinking water**	
1) Safely managed	Drinking water from an improved source that is accessible on premises, available when needed and free from faecal and priority chemical contamination. These include piped water on premises, protected well on premises, bottled water, sachet water, rain water and other treated water.
2) Basic	Drinking water from an improved source (e.g., piped water, borehole, protected well, protected springs) within 30 min round trip, including queuing.
3) Limited	Drinking water from an improved source that requires more than 30 min to collect water.
4) Unimproved	Drinking water from unprotected sources (e.g., unprotected well, unprotected spring).
5) Surface water (no service)	Drinking water collected directly from rivers, lakes, ponds, streams, or canals.
b) **Sanitation**	
1) Safely managed	Use of improved sanitation facility that is not shared with other households and where excreta are safely disposed of or treated on-site. These include flush to piped sewer/septic, VIP latrine, pit latrine with slab on household premises
2) Basic	Use of improved sanitation facility that is not shared with other households. Examples: flush/pour-flush to piped sewer/septic tank, ventilated improved pit latrine.
3) Limited	Use of an improved facility shared with other households.
4) Unimproved	Use of pit latrines without a slab, bucket toilets, hanging toilets, or other unimproved facilities.
5) Open defecation	Disposal of human feces in fields, forests, bushes, open bodies of water, beaches, no facility, or other open spaces.

### Independent variables

Identification and categorization of independent variables was based on empirical evidence^25,37–40^ and the standardized categories within the MIS dataset as shown in Table 2. This categorization was necessary for the smooth running of the logistic regressions.

**Table 2:** Categorization of independent variables.

No.	Independent variables	Categories
1	Age	(1) < 30, (2) 31–39, (3) 40–49, (4) 50–59, (5) ≥ 60)
2	Sex	(1) Male, (2) Female
3	Education level	(1) No education, (2) primary, (3) secondary, (4) Higher
4	Religion	(1) No religion (2) Anglican, (3) Catholic, (5) Muslim, (5) Seventh Day Adventists, (6) Born again
5	Place of residence	(1) Rural, (2) Urban, (3) Refugee settlement
6	Household wealth status	(1) Poor, (2) Middle, (3) Rich
7	Household size	(1) 1– 3, (2) 4– 6, (3) 7+
8	Region of residence	(1) Kampala, (2) North Buganda, (3) South Buganda, (4) Busoga, (5) Bukedi, (6) Bugisu, (7) Teso, (8) Karamoja, (9) Lango, (10) Acholi, (11) West Nile, (12) Bunyoro, (13) Tooro, (14) Ankole, (15) Kigezi
9	Distance to water source	(1) On premises (2) < 19 min, (3) 20**–**29 min, (4) Above 30 min
10	Cluster altitude	(1) < 1000 m, (2) 1001**–**1500 m, (3) above 1500 m

### Data analysis

Four types of analyses were performed. Multicollinearity was assessed using the Variance Inflation Factor (VIF). All predictors showed acceptable VIF values (range: 1.12–2.11), indicating no serious multicollinearity. Descriptive analysis of the data was conducted resulting into summarized statistics, bivariate analysis was used to assess the association between the outcome and the independent variables using the chi-square test, and multivariate ordinal logistic regression modelling was conducted to assess the magnitude of the associations after including controls (Eq. 1).

1 $$\:Log\frac{{P(Y_{i} \le \:j}}{{P(Y_{i} < j}}\: = \alpha \:_{j} + \:\beta \:_{1} X_{{1i}} + \:\beta \:_{{12}} X_{{2i}} + \:\beta \:_{3} X_{{3i}} + \cdots \:\beta \:_{k} X_{{ki}} ,\:{\mathrm{j}} = 1,2, \ldots \:,{\mathrm{J}} - 1$$

where Y_*i*_ represents the ordinal outcome variable (water or sanitation service level) for household *i;*_j_ denotes the category threshold; α_*j*_ are the intercepts (cut-points); X_ki_ are the independent variables; and β_k_ are the estimated regression coefficients. A positive coefficient indicates higher likelihood of being in a higher service level category.

The ordinal logistic regression model was used because household drinking water and sanitation service levels are ordinal, ranging from safely managed, basic, limited, unimproved, to no service. This model appropriately accounts for the natural ordering of the categories, unlike linear or multinomial regression, allowing for the estimation of the effects of demographic and socio-economic factors on the likelihood of households achieving higher versus lower service levels. Post-estimation diagnostics were conducted to assess the adequacy of the ordinal logistic regression models. Model fit was evaluated by comparing the log-likelihoods of the fitted models with those of the intercept-only models and by examining McFadden’s pseudo R². These diagnostics were applied to both the drinking water and sanitation models to confirm model robustness and ensure the reliability of the estimated associations.

The multivariate ordinal logistic results were presented with coefficients with 95% confidence intervals (CI). The *p-values* of < 0.05 were set for statistical significance. The inclusion criteria for variables in the multivariable ordinal logistic regression were set at *P* < 0.05. This threshold was chosen to ensure that only variables with a statistically significant contribution to explaining the outcome were included, thereby enhancing the robustness of the findings. To account for disproportionate sampling and nonresponse, DHS weights provided as six-digit integers in the HR file dataset were normalized (i.e. divided by 1,000,000) and used to provide weights to the data^41^. To adjust for the effect of the complex survey design, the DHS variables of primary sampling unit, strata and sampling weights were used in the analysis. All data entries and statistical analyses were conducted in JMP software, Excel and Stata 18 (Stata Corp, College Station, TX, USA). All methods were performed in accordance with the relevant guidelines and regulations.

## Results

### Categorization of household drinking water and sanitation service levels in Uganda

Table 3, shows that access to drinking water services varied widely across Uganda. Nationally, basic water services accounted for the largest share at 67.8%, with most regions, particularly Busoga, Acholi, Lango, West Nile, and central Uganda showing high dependence on improved sources located within a 30-minute collection time. In contrast, only 8.8% of households had safely managed drinking water, and several regions, including Karamoja, reported no safely managed access. Safely managed services were most common in Kampala and parts of central and western Uganda but remained low overall, indicating limited availability of water on premises that is both accessible and free from contamination. Unimproved sources accounted for 17.0% of households, with the highest concentrations in Acholi, West Nile, Bunyoro, Tooro, and Ankole. Further, 5.9% of the households relied on surface water, especially in Bunyoro, Tooro, Karamoja, and Ankole, underscoring persistent dependence on untreated sources. Only 0.5% of households used limited water services, reflecting a small but notable segment with improved yet time-intensive access.

Sanitation service levels showed a slightly better distribution of improved options but remained constrained by sharing and regional disparities (Table 3). Nationally, 43.6% of households had safely managed sanitation, with higher concentrations in West Nile, Tooro, Ankole, and Acholi. However, limited sanitation, representing improved but shared facilities, constituted 49.7% of households and was widespread across nearly all regions, reflecting the dominant mode of sanitation access. Basic sanitation was extremely low (0.2%), indicating that very few households had private improved facilities. Unimproved sanitation comprised 1.7%, although higher levels were observed in Kigezi, Ankole, and selected eastern regions. Open defecation, while 4.8% nationally, was disproportionately concentrated in Karamoja, where it was the predominant sanitation practice.

Household and contextual characteristics further highlighted regional inequalities in service access (Table 3). Younger household heads were more common in central Uganda, while older household heads were concentrated in eastern and western regions. Female-headed households accounted for 30.4% nationally but exceeded 70% in Karamoja. Educational attainment remained low overall, with 80.1% of households headed by individuals with only primary education. Walking time to water sources showed that 43.7% of households required more than 30 min, especially in Karamoja and several northern regions. Large households (seven or more members) comprised 27.4%, and poverty was widespread, affecting 44.8% of households, particularly in Karamoja, West Nile, and Acholi. Most households (68.6%) resided in rural areas, and 6.8% lived in refugee settlements, primarily in West Nile and Acholi.

**Table 3:** National and regional categorization of drinking water and sanitation service levels (*n* = 8925).

Variables	Region of residence															
	National *n* (%)	Kampala *n* (%)	South Buganda *n* (%)	North Buganda *n* (%)	Busoga *n* (%)	Bukedi *n* (%)	Bugisu *n* (%)	Teso *n* (%)	Karamoja *n* (%)	Lango *n* (%)	Acholi *n* (%)	West Nile *n* (%)	Bunyoro *n* (%)	Tooro *n* (%)	Ankole *n* (%)	Kigezi *n* (%)
**Drinking water service level**																
Safely managed	789 (8.8)	171 (1.9)	164(1.8)	40(0.5)	16(0.2)	9(0.1)	43(0.5)	32(0.34)	0(0.0)	46(0.5)	16(0.2)	9(0.1)	6(0.1)	28(0.3)	49(0.6)	35(0.4)
Basic	6055 (67.8)	305(3.4)	413(4.6)	362(4.1)	411(4.6)	476(5.3)	357(4.0)	446(5.0)	443(5.0)	499(5.6)	538(6.0)	778(8.7)	398(4.5)	453(5.1)	266(3.0)	297(3.3)
Limited	48 (0.5)	0(0.0)	6(0.1)	7(0.1)	4(0.1)	0(0.0)	1(0.0)	12(0.1)	0(0.0)	0(0.0)	0(0.0)	0(0.0)	19(0,2)	0(0.0)	1(0.0)	0(0.0)
Unimproved	1516(17.0)	5(0.1)	95(1.1)	86(1.0)	10(0.1)	12(0.1)	45(0.5)	7(0.1)	4(0.0)	90(1.0)	159(1.8)	108(1.2)	172(1.9)	192(2.3)	135(1.5)	76(0.9)
Surface water	526 (5.9)	0(0.)	34(0.4)	40(0.5)	14(0.16)	1(0.0)	37(0.41)	0(0.0)	36(0.4)	25(0.3)	9(0.1)	16(0.2)	90(1.0)	77(0.9)	115(1.3)	78(0.9)
**Sanitation service level**																
Safely managed	3895(43.6)	420(4.7)	464(5.2)	296(3.3)	148(1.7)	213(2.4)	210(2.4)	106(1.20	18(0.2)	90(1.0)	238(2.7)	419(4.7)	212(2.4)	292(3.3)	268(3.0)	109(1.1)
Basic	16(0.2)	17(0.2)	0(0.0)	0(0.0)	1(0.0)	0(0.0)	0(0.0)	0(0.0)	0(0.0)	0(0.0)	1(0.0)	0(0.0)	0(0.0)	0(0.0)	5(0.1)	0(0.0)
Limited	4441(49.7)	41(0.5)	237(2.7)	233(2.6)	282(3.2)	255(2.9)	269(3.0)	312(3.5)	103(1.2)	544(6.1)	355(4.0)	417(4.7)	446(5.0)	436(4.9)	263(3.0)	330(3.7)
Unimproved	155(1.7)	2(0.0)	4(0.0)	5(0.0)	13(0.2)	8(0.0)	1(0.0)	1(0.0)	0(0.0)	0(0.0)	25(0.3)	4(0.0)	8(0.1)	14(0.2)	28(0.3)	46(0.5)
Open defecation	428(4.8)	1(0.0)	7(0.1)	1(0.0)	11(0.1)	22(0.3)	3(0.0)	78(0.9)	362(4.1)	26(0.3)	103(1.2)	71(0.8)	19(0.2)	8(0.1)	3(0.0)	1(0.0)
**Age of household head**																
Less than 30	2161(24.2)	178(37.0)	163(22.9)	148(27.7)	98(21.5)	131(26.3)	112(23.2)	124(25.0)	191(39.5)	175(26.5)	184(25.5)	265(29.1)	190(27.7)	198(26.4)	108(19.1)	114(23.5)
Between 31–39	1907(21.3)	110(22.9)	179(25.1)	105(19.6)	85(18.7)	92(18.5)	102(21.1)	119(23.9)	91(18.8)	156(23.6)	162(22.4)	197(21.6)	158(23.1)	192(25.6)	116(20.5)	103(21.2)
Between 40 − 49	1904(21.3)	80(16.6)	173(24.3)	105(19.6)	109(24.0)	91(18.3)	96(19.9)	98(19.7)	62(12.8)	138(20.9)	156(21.6)	167(18.3)	152(22.2)	130(17.3)	137(24.2)	98(20.2)
Between 50 − 59	1455(16.3)	79(16.4)	95(13.3)	91(17.0)	69(15.2)	70(14.10	79(16.4)	69(13.9)	60(12.4)	98(14.9)	104(14.4)	132(14.5)	88(12.9)	108(14.4)	105(18.5)	71(14.6)
60 and above	1509(16.9)	34(7.1)	102(14.3)	86(16.1)	94(20.7)	114(22.9)	94(19.5)	87(17.5)	79(16.4)	93(14.1)	116(16.1)	150(16.5)	97(14.20	122(16.30	101917.8)	100(20.6)
**Sex of household head**																
Female	2716(30.4)	164(34.1)	193(27.1)	145(271)	100(22.0)	108(21.7)	90(18.6)	126(25.4)	346(71.6)	173(26.2)	295(40.9)	417(45.8)	158(23.1)	194(25.9)	168(29.6)	117(24.1)
Male	6219(69.6)	317(65.9)	519(72.9)	390(72.9)	355(78.0)	390(78.3)	393(81.4)	371(74.7)	137(28.4)	487(73.8)	427(59.10	494(54.2)	527(76.9)	556(74.1)	399(70.4)	369(75.9)
**Education level**																
No education	699(7.8)	3(0.6)	15(2.1)	23(4.3)	36(8.0)	21(4.2)	13(2.7)	24(4.8)	209(43.3)	55(8.3)	76(10.5)	158(17.3)	58(8.5)	85(11.3)	44(7.8)	40(8.2)
Primary	7155(80.1)	357(74.2)	540(75.8)	430(80.4)	378(83.1)	431(86.6)	399(82.6)	415(83.5)	264(54.7)	571(86.5)	586(81.2)	709(77.8)	546(79.7)	587(78.3)	472(83.3)	404(83.1)
Secondary	880(9.9)	95(19.6)	124(17.4)	73(13.6)	38(8.4)	40(8.0)	55(11.4)	41(8.3)	8(1.7)	26(3.9)	50(6.9)	37(4.1)	68(9.9)	66(8.8)	39(6.9)	32(6.6)
Higher	201(2.2)	26(5.4)	33(4.6)	9(1.7)	3(0.7)	6(1.2)	16(3.3)	17(3.4)	2(0.4)	8(1.2)	10(1.4)	7(0.8)	13(1.9)	12(1.6)	12(2.10	10(2.1)
**Religion of household**																
No religion	73(0.8)	1(0.2)	3(0.4)	691.1)	0	3(0.6)	1(0.2)	0	0	7(1.1)	10(1.4)	6(0.7)	20(2.9)	24(3.2)	6(1.1)	1(0.2)
Anglican	2877(32.4)	128(26.6)	166(23.3)	137(25.60	98(21.50	193(38.8)	248(51.4)	206(41.5)	115(23.8)	242(36.7)	99(14.2)	241(27.4)	240(35.0)	327(43.6)	260(45.9)	251(51,7)
Catholic	3279(36.9)	132(27.4)	295(41.4)	163(30.5)	104(22.9)	169(33.9)	117(24.2)	199(40.0)	318(65.8)	336(50.9)	486(69.9)	432(49.0)	275(40.20	229(30.5)	199(35.1)	122(25.1)
Muslim	1224(13.8)	116(24.1)	134(18.8)	113(21.1)	139(30.6)	73(14.7)	18(3.7)	7(1.4)	9(1.9)	3(0.5)	11(1.6)	123(14.0)	25(3.7)	29(3.9)	17(3.0)	25(5.1)
Seventh-day Adventist (SDA)	148(1.7)	6(1.3)	12(1.7)	25(4.7)	3(0.7)	0	0	0	4(0.8)	3(0.5)	0	10(1.1)	13(1.9)	43(5.7)	11(1.9)	18(3.7)
Born again	1288(14.5)	98(20.37)	102(14.30	91(17.0)	111(24.4)	60(12.1)	99(20.5)	85(17.10	37(7.7)	69(10.5)	89(12.8)	69(7.8)	112(16.40	98(13.1)	74(13.1)	69(14.2)
**Cluster altitude (Meters)**																
Less than 1000	578(6.5)	0	0	0	0	0	0	0	0	0	151(21.6)	616(69.6)	50(7.3)	68(9.4)	0	0
Between 1001 − 1500	7275(82.5)	481(100)	712(100)	535(100)	455(100)	498(100)	309(64.0)	497(100)	403(83.4)	633(100)	547(78.4)	245(27.7)	635(92.7)	575(79.6)	347(61.2)	56(11.5)
1501 and above	967(11.0)	0	0	0	0	0	174(36.0)	0	80(16.6)	0	0	24(2.7)	0	79(10.9)	220(38.8)	430(88.5)
**Walk time distance (minutes)**																
On premises	1805(20.2)	292(61.5)	294(41.3)	129(24.1)	46(10.1)	23(4.6)	66(13.7)	66(13.3)	3(0.6)	107(16.2)	72(10.0)	200(22.0)	53(7.7)	51(6.8)	91(16.1)	95(19.6)
Less than 19	1967(22.0)	154(32.0)	164(23.0)	123(22.9)	88(19.3)	125(25.1)	126(26.1)	77(15.5)	14(3.0)	127(19.2)	132(18.3)	138*15.2)	185(27.0)	209(27.9)	166(29.3)	107(22.0)
Between 20 − 29	1260(14.1)	17(3.53)	60(8.4)	62(11.6)	61(13.4)	122(24.5)	101(20.9)	52(10.5)	48(10.0)	100(15.2)	103(14.3)	149(16.4)	115(16.8)	112(14.9)	104(18.3)	60(12.4)
30 and above	3903(43.7)	14(2.9)	194(27.3)	221(41.30	260(57.1)	228(45.8)	190(39.3)	302(60.8)	418(86.5)	326(49.4)	415(57.5)	424(46.5)	332(48.5)	378(50.4)	206(36.3)	224(46.1)
**Household size**																
Between 1 − 3	3044(34.1)	244(50.7)	269(37.8)	204(38.1)	144(31.65)	176(35.3)	169(35.0)	129(26.0)	142(29.4)	207(31.4)	175(24.2)	227(24.9)	194(28.3)	212(28.3)	197(34.7)	142(29.2)
Between 4 − 6	3447(38.6)	166(34.5)	292(41.0)	212(39.6)	155(34.1)	197(39.6)	177(36.7)	159(32.0)	257(53.2)	272(41.2)	294(40.7)	354(38.9)	282(41.2)	287(38.3)	218(38.5)	232(47.7)
7 and above	2444(27.4)	71(14.8)	151(21.2)	119(22.2)	156(34.3)	125(25.1)	137(28.4)	209(42.0)	84(17.4)	181(27.4)	253(35.0)	330(36.2)	209(30.5)	251(33.5)	152(26.8)	112(23.1)
**Household wealth status**																
Poor	3999(44.8)	0	91(12.8)	140(26.17	185(40.7)	284(57.0)	207(42.9)	354(71.2)	470(97.3)	472(71.5)	558(77.3)	768(84.3)	331(48.30	328(43.7)	212(37.4)	201(41.4)
Middle	1586(17.8)	2(0.4)	119(16.7)	113(21.1)	112(24.6)	101(20.3)	109(22.6)	32(6.4)	4(0.8)	66(10.0)	59(8.2)	67(7.4)	143(20.9)	219(29.2)	103(18.2)	148(30.5)
Rich	3349(37.5)	479(99.6)	502(70.5)	282(52.70	158(34.70	113(22.70	167(34.6)	111(22.3)	9(1.9)	122(18.5)	105(14.5)	76(8.3)	211(30.8)	203(27.1)	252(44.4)	137(28.2)
**Place of residence**																
Rural	6130(68.6)	0	327(45.9)	387(72.30	348(76.5)	425(85.3)	350(72.5)	424(85.3)	456(94.4)	554(83.9)	544(75.4)	502(55.1)	558(81.5)	574(76.5)	350(61.7)	378(77.8)
Urban	2201(24.6)	481(100)	385(54.1)	148(27.7)	107(23.5)	73(14.7)	133(27.5)	73(14.70	27(5.6)	106(16.1)	151(20.9)	26(2.9)	101(14.7)	92(12.3)	134(23.6)	108(22.2)
Refugee settlements	604(6.8)	0	0	0	0	0	0	0	0	0	27(3.74)	383(42.0)	26(3.8)	84(11.2)	83(14.6)	0

### Categorization of household drinking water and sanitation service levels by type of residence

Table 4, shows that access to drinking water and sanitation services varied markedly by residence type, demonstrating substantial structural inequities between urban, rural, and refugee settlement populations. Safely managed drinking water was highest in urban areas, where 5.5% of households met this standard, compared with 2.0% in rural areas and only 0.3% in refugee settlements. The majority of urban and rural households relied on basic water services, though the burden was considerably heavier in rural areas, where 50.0% depended on basic services versus 15.9% in urban settings. Refugee settlements showed the greatest reliance on basic water services (6.3%), but also exhibited disproportionately high dependence on surface water (0.3%) relative to their population size, reflecting limited access to improved sources. Unimproved water sources were predominantly rural (11.3%), while urban households showed the lowest reliance (2.0%), and refugee settlements remained vulnerable, with 1.1% depending on unimproved sources.

Patterns in sanitation access aligned closely with those of drinking water, but with even sharper disparities (Table 4). Urban areas showed the highest share of safely managed sanitation services (15.9%), followed by rural areas (19.6%) and refugee settlements (3.4%). Rural households, however, were overwhelmingly reliant on limited sanitation services at 41.2%, compared with 6.9% in urban areas and 2.5% in refugee settlements. Refugee settlements showed the lowest access to safely managed sanitation and remained disproportionately dependent on inadequate options, including open defecation, which affected 3.2% of households. Open defecation was most prevalent in rural homes (7.1%), indicating persistent sanitation deficits in non-urban regions. Although urban areas demonstrated comparatively better access to improved sanitation, open defecation remained present (0.6%), highlighting that even urban settings retain pockets of deprivation.

**Table 4:** **Categorization of drinking water and sanitation service level by type of residence**.

Variables	Urban (2,145, 24.0%)	Rural (6,177, 69.2%)	Refugee settlements (603, 6.8%)
**Drinking water service levels**			
Safely managed	486(5.5)	175(2.0)	3(0.0)
Basic	1,419(15.9)	4,458(50.0)	566(6.3)
Limited	19(0.2)	31(0.4)	0(0.0)
Unimproved	181(2.0)	1,004(11.3)	11(0.1)
Surface water	40(0.5)	509(5.7)	23(0.3)
**Sanitation service levels**			
Safely managed	1,419(15.9)	1,746(19.6)	338(3.4)
Basic	21(0.2)	0(0.0)	3(0.0)
Limited	616(6.9)	3,680(41.2)	227(2.5)
Unimproved	35(0.4)	118(1.3)	6(0.0)
Open defecation	54(0.6)	633(7.1)	29(0.32)

### Factors associated with drinking water service levels in Uganda

Analysis of the association between the independent variables and drinking water service levels revealed that the majority of the examined factors were significantly associated with the type of water service accessed by households (Table 5). Out of the ten variables included in the model, eight demonstrated statistically significant associations (*p* < 0.05). Significant predictors included sex of household head, education level, cluster altitude, walk-time distance to water sources, region of residence, household size, household wealth status, and place of residence. These variables showed varying relationships with safely managed, basic, limited, unimproved, and surface water service levels. In contrast, age of the household head and religion did not show statistically significant associations (*p* > 0.05), indicating limited influence on household drinking water service levels in the context of this dataset. Overall, the results highlight substantial socio-demographic and spatial disparities in access to improved drinking water services across households in Uganda.

**Table 5:** *Statistical association between the independent variables and the drinking water serve levels*.

Independent variables	Dependent variable: Drinking water serve levels										
	Safely managed		Basic		Limited		Unimproved		Surface water		*P-values*
	%	CI	%	CI	%	CI	%	CI	%	CI	
**Age of household head**											0.189
Less than 30	9.4	[7.1,12.3]	71.8	[67.2,76.0]	0.8	[0.3,2.2]	12.6	[8.8,17.7]	5.4	[3.8,7.5]	
Between 31**–**39	8.9	[6.1,12.6]	65.9	[56.8,73.9]	0.6	[0.2,1.5]	18.3	[10.6,29.8]	6.3	[4.4,9.0]	
Between 40– 49	7.7	[5.3,10.9]	67.3	[57.7,75.7]	0.3	[0.1,1.0]	18.4	[10.4,30.4]	6.4	[4.5,9.0]	
Between 50**–**59	10.2	[7.1,14.5]	62.5	[49.2,74.2]	0.2	[0.1,0.5]	21.8	[10.2,40.8]	5.3	[3.4,8.0]	
60 and above	8.2	[5.9,11.3]	70	[62.9,76.2]	0.8	[0.3,1.8]	15.1	[10.6,21.1]	5.9	[4.2,8.3]	
**Sex of household head**											0.03*
Female	8.9	[6.6,11.9]	68.3	[60.7,75.0]	0.6	[0.2,1.4]	17.1	[10.7,26.2]	5.1	[3.6,7.3]	
Male	8.8	[6.7,11.5]	67.5	[59.8,74.4]	0.5	[0.3,1.0]	16.9	[10.2,26.8]	6.2	[4.5,8.5]	
**Highest education level**											< 0.01*
No education	1.4	[0.6,2.9]	75.2	[67.9,81.3]	0		16.4	[10.9,24.0]	7	[4.6,10.3]	
Primary	8.4	[6.5,10.9]	67.4	[59.8,74.2]	0.6	[0.3,1.2]	17.4	[10.8,26.9]	6.2	[4.5,8.3]	
Secondary	13.6	[9.5,19.0]	65.9	[55.3,75.1]	0.2	[0.1,0.7]	16.5	[7.6,32.4]	3.9	[2.0,7.2]	
Higher	28.9	[21.3,37.8]	64.5	[56.4,71.9]	0.3	[0.1,1.6]	5	[2.2,10.9]	1.2	[0.3,4.6]	
**Religion of the household**											0.544
No religion	5.3	[1.5,17.1]	59.1	[41.1,75.0]	0.4	[0.0,2.6]	22.4	[11.3,39.4]	12.9	[5.8,26.3]	
Anglican	7.9	[5.6,11.0]	67.7	[61.0,73.8]	0.5	[0.3,1.1]	15.9	[10.8,23.0]	7.9	[5.5,11.3]	
Catholic	9.1	[7.1,11.7]	67.7	[61.5,73.3]	0.5	[0.2,1.2]	17.6	[12.4,24.3]	5.1	[3.6,7.0]	
Muslim	10.4	[6.5,16.1]	65.5	[46.8,80.4]	0.9	[0.2,4.8]	19.6	[6.2,47.5]	3.6	[1.6,7.7]	
Seventh-day Adventist	6.8	[3.1,14.6]	65.1	[53.9,74.8]	0		19.6	[11.6,31.1]	8.5	[4.5,15.3]	
Born again	9.5	[6.0,14.5]	69.9	[60.8,77.7]	0.3	[0.1,1.2]	15.2	[8.3,26.1]	5.1	[3.4,7.7]	
**Cluster altitude (Meters)**											<0.01*
Less than 1000	1.1	[0.4,3.3]	90.7	[81.2,95.7]	0.4	[0.0,2.5]	4.6	[1.9,11.0]	3.1	[1.0,9.2]	
Between 1001**–**1500	10	[7.5,13.1]	66.5	[57.7,74.3]	0.6	[0.3,1.2]	18	[10.3,29.5]	5	[3.4,7.1]	
1500 and above	6	[3.3,10.7]	61.5	[48.0,73.5]	0		17.3	[11.3,25.5]	15.2	[8.6,25.4]	
**Walk time distance (minutes)**											< 0.01*
On premises	40.5	[31.0,50.6]	45.4	[37.1,54.0]	1.3	[0.5,3.0]	11.7	[3.0,36.0]	1.2	[0.5,2.8]	
Less than 19	1.7	[1.0,2.8]	74	[67.6,79.6]	0.8	[0.3,2.2]	15	[10.8,20.6]	8.4	[5.5,12.6]	
Between 20**–**29	1	[0.2,6.0]	72	[62.6,79.8]	0.3	[0.1,1.6]	19.8	[12.5,29.9]	6.9	[4.7,10.0]	
30 and above	0.3	[0.1,0.8]	73.6	[65.5,80.4]	0.1	[0.1,0.3]	19.5	[13.0,28.2]	6.5	[4.5,9.2]	
**Region of residence**											< 0.01*
Kampala	40.3	[28.7,53.0]	58.7	[46.4,70.0]	0		1	[0.4,2.8]	0		
South Buganda	22.6	[14.5,33.6]	56.8	[41.7,70.7]	0.9	[0.2,3.5]	15.7	[6.6,32.8]	4	[1.5,10.2]	
North Buganda	5	[2.5,9.7]	55.4	[25.2,82.1]	1.3	[0.4,4.2]	34.3	[8.9,73.4]	4.1	[1.3,12.3]	
Busoga	3.9	[0.8,16.5]	88.2	[72.2,95.6]	0.9	[0.4,2.3]	2	[0.8,4.6]	4.9	[0.9,23.9]	
Bukedi	1.7	[0.4,6.9]	96	[92.1,98.0]	0		2.2	[1.0,4.6]	0.1	[0.0,0.9]	
Bugisu	8.3	[2.7,23.1]	69.4	[46.2,85.7]	0.1	[0.0,0.5]	9.2	[3.4,22.3]	13	[4.0,35.2]	
Teso	7.7	[1.3,34.7]	90.7	[67.2,97.9]	0.7	[0.1,5.0]	0.9	[0.2,3.7]	0		
Karamoja	0		92.7	[84.9,96.6]	0		1.2	[0.2,7.7]	6.2	[2.6,13.8]	
Lango	7.6	[2.9,18.7]	77	[64.7,86.0]	0		13.1	[6.3,25.3]	2.2	[0.8,6.1]	
Acholi	2.6	[1.0,6.8]	74.6	[60.9,84.7]	0		21.2	[11.7,35.3]	1.5	[0.2,9.1]	
West Nile	1	[0.3,3.4]	82.5	[72.6,89.4]	0		14.4	[8.5,23.3]	2	[0.9,4.5]	
Bunyoro	0.8	[0.3,2.1]	60.2	[47.8,71.5]	2.1	[0.7,5.9]	27.4	[18.6,38.4]	9.5	[4.3,19.8]	
Tooro	2.5	[1.4,4.4]	61.2	[49.6,71.7]	0		28.6	[19.6,39.7]	7.7	[3.6,15.5]	
Ankole	8.2	[4.4,14.8]	51.8	[38.2,65.1]	0.1	[0.0,1.0]	21.8	[12.8,34.6]	18.1	[11.9,26.4]	
Kigezi	7.7	[4.2,14.0]	64.6	[52.1,75.3]	0		13.7	[7.2,24.5]	13.9	[7.0,25.9]	
**Household size**											< 0.01*
Between 1**–**3	12.6	[9.5,16.6]	63.3	[53.8,71.8]	1.3	[0.7,2.5]	17.8	[9.3,31.5]	5	[3.5,7.1]	
Between 4– 6	6.2	[4.7,8.2]	70.4	[63.0,76.9]	0.2	[0.1,0.5]	17.2	[10.8,26.2]	6	[4.4,8.0]	
7 and above	7.8	[5.2,11.5]	69.7	[63.4,75.2]	0	[0.0,0.1]	15.7	[11.2,21.4]	6.9	[4.5,10.3]	
**Household wealth status**											< 0.01*
Poor	0.6	[0.3,1.1]	71.2	[60.8,79.7]	0.4	[0.2,0.8]	20.5	[12.2,32.5]	7.4	[5.2,10.3]	
Middle	2.7	[1.6,4.5]	70.8	[63.4,77.2]	0.9	[0.3,2.9]	18.5	[12.9,25.7]	7.1	[4.7,10.6]	
Rich	21.6	[17.5,26.4]	62.3	[55.4,68.7]	0.6	[0.3,1.1]	12	[6.8,20.3]	3.5	[2.4,5.1]	
**Place of residence**											< 0.01*
Rural	3.6	[2.6,5.0]	67.5	[56.9,76.5]	0.7	[0.3,1.3]	21	[12.0,33.9]	7.3	[5.2,10.2]	
Urban	25.7	[20.1,32.2]	62.9	[55.1,70.1]	0.3	[0.1,0.8]	9.2	[5.5,14.9]	1.9	[0.8,4.4]	
Refugee settlements	0.7	[0.2,2.5]	88.7	[73.5,95.7]	0		4.9	[1.0,21.5]	5.8	[1.6,19.0]	

* P-value significant at 0.05, %=percentage, CI = Confidence interval.

### Factors associated with sanitation service levels in Uganda

In Table 6, all examined independent variables showed statistically significant associations with sanitation service levels (*p* < 0.05). The results indicate clear socio-demographic and spatial disparities in access to safely managed sanitation.

**Table 6:** Statistical association between the independent variables and the sanitation serve levels.

Independent variables	Dependent variable: Sanitation serve levels										
	Safely managed		Basic		Limited		Unimproved		Open defecation		P-values
	%	CI	%	CI	%	CI	%	CI	%	CI	
**Age of household head**											< 0.01*
Less than 30	47.3	[41.7,53.0]	0.3	[0.1,0.8]	44.2	[39.0,49.6]	1.3	[0.8,2.2]	6.8	[5.4,8.5]	
Between 31**–**39	44.5	[38.9,50.3]	0.1	[0.0,0.5]	49.6	[43.7,55.5]	1.9	[1.2,2.9]	3.8	[2.9,5.1]	
Between 40– 49	42	[37.1,47.1]	0.1	[0.0,0.4]	51.5	[46.2,56.7]	2.4	[1.3,4.6]	4.0	[3.0,5.3]	
Between 50**–**59	42.4	[36.4,48.7]	0.3	[0.1,1.0]	51.7	[44.9,58.4]	1.4	[0.8,2.4]	4.2	[3.0,5.8]	
60 and above	40.1	[35.4,45.1]	0	[0.0,0.1]	53.5	[48.8,58.2]	1.5	[0.9,2.7]	4.8	[3.6,6.3]	
**Sex of household head**											< 0.01*
Female	48.5	[43.5,53.6]	0.3	[0.1,0.6]	41.3	[36.6,46.2]	1.4	[0.9,2.4]	8.4	[6.7,10.6]	
Male	41.4	[37.0,46.0]	0.1	[0.1,0.3]	53.4	[48.6,58.1]	1.9	[1.2,2.8]	3.2	[2.5,4.1]	
**Highest education level**											< 0.01*
No education	30.2	[23.3,38.2]	0.3	[0.0,1.8]	53.3	[45.8,60.7]	1.5	[0.8,2.6]	14.8	[11.2,19.3]	
Primary	41.5	[37.8,45.4]	0.1	[0.1,0.3]	52.1	[48.1,56.1]	1.8	[1.2,2.9]	4.4	[3.6,5.4]	
Secondary	62	[53.1,70.1]	0.4	[0.1,1.5]	35.4	[27.3,44.3]	1.6	[0.8,3.2]	0.7	[0.3,1.5]	
Higher	82.6	[73.0,89.2]	0.8	[0.1,5.8]	14.6	[8.9,22.8]	0.4	[0.1,2.7]	1.7	[0.3,9.2]	
**Religion of the household**											0.02*
No religion	38.6	[22.3,57.9]	0		57.6	[38.7,74.5]	1.6	[0.2,10.7]	2.2	[0.5,8.5]	
Anglican	41.6	[37.3,45.9]	0.2	[0.1,0.4]	51.8	[47.5,56.2]	2.3	[1.3,4.2]	4.1	[3.0,5.6]	
Catholic	41.8	[37.4,46.3]	0.2	[0.1,0.6]	49.7	[45.3,54.1]	1.4	[0.9,2.2]	6.9	[5.5,8.7]	
Muslim	48.3	[36.9,59.8]	0.2	[0.1,0.9]	48.1	[36.1,60.3]	1.2	[0.6,2.5]	2.3	[1.2,4.1]	
Seventh-day Adventist	43.2	[31.6,55.5]	0		47.1	[33.6,61.0]	6.9	[1.6,25.6]	2.9	[0.7,10.6]	
Born again	47.6	[42.1,53.2]	0.2	[0.0,0.7]	47.3	[41.9,52.8]	1.4	[0.8,2.3]	3.5	[2.4,5.1]	
**Cluster altitude (Meters)**											< 0.01*
Less than 1000	53	[42.0,63.8]	0		36.9	[27.4,47.6]	1.8	[0.7,4.3]	8.3	[5.3,12.6]	
Between 1001**–**1500	44.3	[39.4,49.4]	0.2	[0.1,0.4]	49.7	[44.5,54.9]	1	[0.7,1.6]	4.7	[3.7,6.0]	
1500 and above	33.7	[26.0,42.4]	0		56.6	[47.3,65.5]	6.9	[3.0,14.8]	2.9	[1.2,6.5]	
**Walk time distance (minutes)**											< 0.01*
On premises	70.2	[61.7,77.6]	0.6	[0.3,1.1]	26.4	[18.9,35.4]	0.6	[0.2,1.5]	2.2	[1.4,3.6]	
Less than 19	47.6	[41.2,54.0]	0.1	[0.0,0.4]	46.8	[40.7,53.0]	3	[1.7,5.4]	2.5	[1.6,3.9]	
Between 20**–**29	36.6	[32.3,41.0]	0.1	[0.0,0.8]	57.2	[52.6,61.7]	1.7	[0.9,3.3]	4.5	[3.0,6.5]	
30 and above	31.5	[27.8,35.5]	0.1	[0.0,0.5]	59.5	[55.6,63.4]	1.6	[1.1,2.3]	7.2	[5.8,9.0]	
**Region of residence**											< 0.01*
Kampala	86.9	[78.4,92.3]	2.3	[1.2,4.5]	10	[5.3,18.0]	0.3	[0.1,1.3]	0.5	[0.1,3.0]	
South Buganda	65.6	[51.3,77.6]	0		33.2	[21.6,47.3]	0.3	[0.1,1.0]	0.9	[0.3,2.4]	
North Buganda	46	[30.5,62.2]	0		52.9	[36.1,69.1]	1	[0.3,3.3]	0.2	[0.0,1.2]	
Busoga	33.4	[21.7,47.7]	0.3	[0.0,1.8]	60.7	[47.6,72.3]	3.2	[1.6,6.5]	2.4	[1.4,4.3]	
Bukedi	40.9	[31.8,50.7]	0		52.6	[43.5,61.5]	1.8	[0.8,4.0]	4.7	[3.1,7.2]	
Bugisu	46.5	[31.2,62.6]	0		52.5	[36.3,68.3]	0.2	[0.0,1.4]	0.7	[0.2,2.4]	
Teso	19.3	[9.1,36.2]	0		63.3	[50.4,74.5]	0.2	[0.0,1.2]	17.3	[9.9,28.5]	
Karamoja	2.5	[0.8,7.6]	0		19.5	[10.9,32.5]	0		78.0	[63.4,87.9]	
Lango	15	[7.9,26.7]	0		80.9	[70.7,88.1]	0		4.1	[2.5,6.5]	
Acholi	38.8	[25.5,54.0]	0.2	[0.0,1.4]	43.1	[31.5,55.5]	3	[1.2,7.3]	14.9	[8.8,24.2]	
West Nile	44.3	[34.0,55.2]	0		46.2	[36.4,56.3]	0.5	[0.2,1.5]	9.0	[6.5,12.5]	
Bunyoro	30.9	[23.8,39.1]	0		66	[58.3,72.9]	0.7	[0.3,1.6]	2.4	[1.1,5.2]	
Tooro	38.7	[25.5,53.7]	0		57.2	[42.1,71.1]	2.1	[0.8,5.1]	2.1	[0.9,4.5]	
Ankole	46.6	[38.1,55.3]	0.7	[0.2,2.7]	46.6	[36.8,56.6]	5.7	[1.8,16.8]	0.4	[0.1,1.7]	
Kigezi	22.1	[16.4,29.1]	0		68.2	[59.9,75.5]	9.4	[4.8,17.7]	0.3	[0.0,1.9]	
**Household size**											< 0.01*
Between 1**–**3	46.1	[40.4,51.9]	0.2	[0.1,0.6]	46.2	[40.0,52.4]	2.2	[1.2,3.9]	5.4	[4.2,6.9]	
Between 4– 6	42.5	[38.5,46.5]	0.1	[0.0,0.3]	50.3	[46.2,54.4]	1.6	[1.0,2.4]	5.5	[4.5,6.8]	
7 and above	42.1	[37.6,46.7]	0.2	[0.1,0.6]	53.2	[48.7,57.8]	1.4	[0.9,2.3]	3.0	[2.2,4.2]	
**Household wealth status**											< 0.01*
Poor	20.7	[16.7,25.3]	0.1	[0.0,0.5]	66.9	[61.3,72.0]	2.1	[1.4,3.2]	10.3	[8.4,12.5]	
Middle	34.1	[29.9,38.5]	0		62.7	[58.4,66.8]	2.2	[1.4,3.6]	1.0	[0.5,1.9]	
Rich	75.4	[71.3,79.2]	0.4	[0.2,0.8]	23	[19.4,27.1]	1.1	[0.5,2.4]	0.1	[0.0,0.3]	
**Place of residence**											< 0.01*
Rural	32.3	[28.7,36.2]	0		59.7	[55.5,63.8]	2	[1.2,3.3]	5.9	[4.6,7.5]	
Urban	70.8	[62.7,77.9]	0.6	[0.3,1.2]	25.9	[19.5,33.6]	1.1	[0.5,2.4]	1.5	[0.6,3.6]	
Refugee settlements	58.5	[44.7,71.1]	0.4	[0.1,3.2]	34.6	[22.1,49.6]	0.9	[0.3,2.7]	5.5	[3.5,8.6]	

* P-value significant at 0.05, %=percentage, CI = Confidence interval.

### Magnitude of association between the independent variables and the drinking water and sanitation service levels

In order to determine the magnitude of association between the independent and the dependent variables (drinking water and sanitation service levels), an ordinal logistic regression was performed and the results are presented in Tables 7 and 8. The ordinal logistic regression analysis provides important insights into how household and contextual characteristics influence progression across the established drinking water and sanitation service levels in Uganda. Overall, positive coefficients indicate a higher likelihood of households accessing better service levels, while negative coefficients signal a greater likelihood of remaining in lower-quality service levels.

### Drinking water service levels factors

In Table 7, households headed by males demonstrated higher likelihood of accessing higher water service levels (β = 1.115) compared with female-headed households. Educational attainment showed a more pronounced effect. Households with higher education were significantly more likely to move upward along the service ladder (β = 1.618), suggesting that education enhances the ability to access safer and more reliable water options. Walking distance to the household’s water source emerged as a strong determinant of service level. Households located within 19 min of a water source (β = 2.123) and those between 20 and 29 min (β = 2.143) had substantially higher odds of accessing better service levels relative to households whose water was on the premises. These positive coefficients reflect the fact that many improved water supplies in Uganda, such as boreholes or public taps, are typically off premises. In contrast, households walking 30 min or more recorded a negative coefficient (β = − 2.092), indicating significantly lower likelihood of accessing improved or safely managed water and a greater likelihood of relying on limited, unimproved, or surface water sources. Regional disparities remain pronounced. Households in South Buganda (β = 1.579), North Buganda (β = 1.434), West Nile (β = 0.739), Bunyoro (β = 1.317), Tooro (β = 1.289), and Ankole (β = 1.647) demonstrated higher likelihoods of accessing improved water services. Conversely, households in Bukedi (β = − 0.752), Teso (β = − 0.954), and Karamoja (β = − 0.923) had substantially lower likelihood of moving into higher service levels, reflecting regional inequalities in water infrastructure and service provision.

Household economic status also played a critical role. Middle-income households (β = 0.578) and especially wealthy households (β = 1.155) were more likely to access higher water service levels compared with poor households, highlighting the importance of economic capacity in securing improved water access. Regarding place of residence, households located in urban areas (β = 1.676) had significantly greater likelihood of accessing higher service levels, consistent with the concentration of piped networks and safely managed services in urban centers. In contrast, households in refugee settlements (β = − 1.249) were far less likely to ascend to higher service levels and more likely to rely on limited or unimproved water sources.

**Table 7:** Ordinal logistic regression analysis for the magnitude of association between the independent variables and household’s water service levels in Uganda.

Independent variables	Coefficient	std. err.	t	*P*>|t|	[95% C.I]
**Sex of the household head**					
Male	1.115	0.098	-1.18	0.04*	[-0.31,0.08]
**Highest education level**					
Primary	-0.208	0.135	-1.55	0.12	[-0.47,0.06]
Secondary	0.121	0.170	-0.71	0.48	[-0.46,0.21]
Higher	1.618	0.287	-2.15	0.03*	[-1.18,0.05]
**Walk time distance (minutes)**					
Less than 19	2.123	0.801	2.65	< 0.01*	[0.56,3.690]
Between 20**–**29	2.143	0.778	2.76	< 0.01*	[0.61,3.671]
30 and above	-2.092	0.808	2.59	< 0.01*	[0.50,3.638]
**Region of residence**					
South Buganda	1.579	0.305	1.90	0.04*	[-0.02,1.178]
North Buganda	1.434	0.800	1.79	0.04*	[-0.141,3.01]
Busoga	-0.115	0.393	-0.29	0.77	[-0.888,0.66]
Bukedi	-0.752	0.361	-2.09	0.03*	[-1.461,0.042]
Bugisu	0.520	0.547	0.95	0.34	[-0.556,1.597]
Teso	-0.954	0.324	-2.90	< 0.01*	[-1.600,0.307]
Karamoja	-0.923	0.424	-2.18	0.03*	[-1.758,-0.089]
Lango	-0.253	0.371	-0.68	0.49	[-0.983,0.477]
Acholi	0.481	0.385	1.25	0.21	[-0.277,1.238]
West Nile	0.739	0.392	1.88	0.04*	[-0.033,1.511]
Bunyoro	1.317	0.415	3.17	< 0.01*	[0.500,2.133]
Tooro	1.289	0.405	3.18	< 0.01*	[0.493,2.087]
Ankole	1.647	0.542	3.04	< 0.01*	[0.580,2.713]
Kigezi	0.914	0.686	1.33	0.18	[-0.436,2.263]
**Household size**					
Between 4**–**6	0.178	0.109	1.64	0.10	[-0.036,0.391]
7 and above	0.169	0.107	1.52	0.13	[-0.048,0.372]
**Household wealth status**					
Middle	0.578	0.152	-3.80	< 0.01*	[-0.878,0.279]
Rich	1.155	0.162	-7.13	< 0.01*	[-1.474,0.837]
**Place of residence**					
Urban	1.676	0.323	-2.10	0.03*	[-1.312,0.416]
Refugee settlements	-1.249	0.273	-4.55	< 0.01*	[-1.789,0.709]

* P-value significant at 0.05, std. err.= standard error, t = t-statistic, CI = Confidence interval.

### Sanitation service levels factors

The ordinal logistic regression analysis revealed significant gradients in sanitation service levels across household demographic, socioeconomic, and spatial characteristics (Table 8). Age of the household head showed a clear upward progression, with households headed by individuals aged 31–39 (β = 1.198), 40–49 (β = 2.343), and 50–59 (β = 3.248) increasingly more likely to fall within higher sanitation service levels compared with the youngest group. This pattern suggests that older household heads may prioritize or have better resources for improved sanitation. Male-headed households also exhibited a positive association with sanitation access (β = 1.297), indicating a greater likelihood of accessing improved or safely managed facilities compared with female-headed households. Education demonstrated a strong influence on sanitation outcomes. Households headed by individuals with secondary education (β = 1.289) and particularly those with higher education (β = 1.807) were more likely to occupy higher sanitation service levels. This underscores the role of education in promoting sanitation awareness, investment, and adoption of improved technologies.

Walking distance to sanitation facilities also influenced service levels. Households located less than 19 min from a sanitation option had a strong positive association (β = 1.463), and those at a distance of 20–29 min (β = 0.676) also tended toward higher service levels, though to a lesser extent. However, households walking 30 min or more to a water source showed a negative association (β = − 0.657), suggesting that long distances impede access to improved sanitation and increase reliance on unimproved or open defecation options. Regional differences were substantial. Households in Busoga (β = 0.694), Teso (β = 1.315), Karamoja (β = 4.399), Lango (β = 0.909), and Kigezi (β = 1.364) showed positive coefficients, indicating a higher likelihood of accessing improved sanitation. Notably, the very high coefficient for Karamoja reflects substantial regional improvements or service concentration, distinguishing it significantly from other regions.

Socioeconomic status strongly shaped sanitation outcomes. Middle-income households (β = 1.897) and rich households (β = 2.363) exhibited markedly higher tendencies toward higher sanitation service levels, demonstrating the critical role of economic capacity in securing improved and safely managed sanitation. Spatial characteristics also played a role. Households in urban areas (β = 1.452) were more likely to access higher sanitation service levels than rural households, reflecting better infrastructure and greater service availability in urban settings. In contrast, households in refugee settlements (β = − 1.856) had a substantially lower likelihood of accessing improved sanitation, pointing to persistent service challenges and infrastructural limitations in these locations.

**Table 8:** Ordinal logistic regression analysis for the magnitude of association between the independent variables and household’s access to sanitation service levels in Uganda.

Independent variables	Coefficient	std. err.	t	*P*>|t|	[95% C.I]
**Age of household head**					
Between 31–39	1.198	0.107	1.85	0.04*	[-0.012,0.410]
Between 40– 49	2.343	0.114	3.00	< 0.01*	[0.118,0.568]
Between 50**–**59	3.248	0.099	2.49	< 0.01*	[0.052,0.443]
60 and above	0.177	0.098	1.80	0.073	[0-0.017,0.372]
**Sex of the household head**					
Male	1.297	0.084	3.52	< 0.01*	[0.131,0.463]
**Highest education level**					
Primary	-0.121	0 0.128	-0.94	0.35	[-0.374,0.133]
Secondary	1.289	0.165	-1.76	0.04*	[-0.614,0.0347]
Higher	1.807	0.308	-2.62	< 0.01*	[-1.414,0.200]
**Religion of the household**					
Anglican	-0.081	0.389	-0.21	0.84	[-0.848,0.686]
Catholic	-0.143	0.393	-0.36	0.72	[-0.917,0.631]
Muslim	-0.250	0.387	-0.65	0.52	[-1.011,0.511]
Seventh-day Adventist	0.158	0.512	0.31	0.76	[-0.849,1.166]
Born again	-0.231	0.400	-0.58	0.56	[-1.018,0 0.556]
**Walk time distance (minutes)**					
Less than 19	1.463	0.128	3.61	< 0.01*	[0.211,0.715]
Between 20**–**29	0.676	0.128	5.30	< 0.01*	[0.424,0.927]
30 and above	-0.657	0 0.112	5.85	< 0.01*	[0.436,0.878]
**Region of residence**					
South Buganda	0.027	0.379	0.07	0.94	[-0.719,0.774]
North Buganda	0.139	0.403	0.35	0.73	[-0.653,0.932]
Busoga	0.694	0.400	1.73	0.04*	[-0.094,1.481]
Bukedi	-0.176	0.410	-0.43	0.67	[-0.983,0.631]
Bugisu	-0.168	0.426	-0.40	0.69	[-1.007,0.669]
Teso	1.315	0.451	2.92	< 0.01*	[0.428,2.201]
Karamoja	4.399	0.581	7.57	< 0.01*	[3.257,5.543]
Lango	0.909	0 0.366	2.48	< 0.01*	[0.189,1.629]
Acholi	0.749	0.517	1.45	0.15	[-0.266,1.766]
West Nile	0.283	0.501	0.57	0.57	[-0.703,1.269]
Bunyoro	0.598	0.408	1.46	0.14	[-0.205,1.401]
Tooro	0 0.243	0.427	0.57	0.57	[-0.598,1.083]
Ankole	0.540	0.382	1.41	0.16	[-0.211,1.292]
Kigezi	1.364	0.462	2.95	< 0.01*	[0.456,2.273]
**Household size**					
Between 4–6	0.018	0.077	0.23	0.82	[-0.133,0.169]
7 and above	-0.127	0 0.101	-1.26	0.21	[-0.327,0.072]
**Household wealth status**					
Middle	1.897	0 0.128	-7.00	< 0.01*	[-1.149,0.645]
Rich	2.363	0.172	-13.7	< 0.01*	[-2.121,2.250]
**Place of residence**					
Urban	1.452	0.165	-2.75	< 0.01*	[-0.776,-0.128]
Refugee settlements	-1.856	0.352	-5.27	< 0.01*	[-2.549,-1.162]

P-value significant at 0.05, std. err.= standard error, t = t-statistic, CI = Confidence interval.

### Model Goodness-of-Fit (Water and Sanitation)

Post-estimation diagnostics indicated that both ordinal logistic regression models demonstrated satisfactory goodness-of-fit. For the drinking water service model, the log-likelihood improved substantially from − 7988.03 in the null model to − 6396.44 in the fitted model, with a McFadden’s pseudo R² of 0.199, indicating acceptable explanatory power. Similarly, the sanitation service model showed strong model performance, with the log-likelihood improving from − 8787.53 (null model) to − 6534.15 in the fitted model and a McFadden’s pseudo R² of 0.256, reflecting robust model fit and explanatory capacity. Overall, these results demonstrate that the fitted models adequately explained variation in water and sanitation service levels and provided a substantial improvement over intercept-only models.

## Discussion

This study provides the first national-level assessment of drinking water and sanitation service levels in Uganda using the WHO/UNICEF Joint Monitoring Programme (JMP) classification, combined with ordinal logistic regression to examine their key determinants. The findings reveal significant disparities across regions, settlement types, and socioeconomic groups, highlighting persistent structural barriers to achieving safely managed services. These results mirror broader patterns documented across sub-Saharan Africa (SSA), where inequalities in access remain a central obstacle to meeting SDG 6 targets.

### Drinking water service levels

The results reveal substantial disparities in drinking water service levels across Uganda, a pattern widely echoed in recent WASH scholarship across sub-Saharan Africa. The predominance of basic drinking water services (67.8%) aligns with findings from Kampala-based studies by Tumwebaze et al.^25^, which similarly reported heavy reliance on improved but off-premises water sources and very limited access to safely managed services in both slum and peri-urban settings. These findings are further supported by the 2000–2024 WHO/UNICEF Progress on Household Drinking Water, Sanitation and Hygiene reports, which note that safely managed drinking water coverage in SSA remains among the lowest globally, with most households dependent on basic or limited services^28,35^. This persistent reliance reflects broader structural challenges faced by many SSA countries, including Uganda, in expanding piped, on-premises, and quality-controlled water systems to all households. The extremely low share of safely managed services nationally (8.8%), and their near absence in regions such as Karamoja, is consistent with regional analyses indicating slow progress in Eastern and Southern Africa, particularly in rural and semi-arid areas where infrastructural expansion is costly and logistically demanding^18^. Conversely, the concentration of safely managed services in Kampala and parts of central and western Uganda supports evidence that urban centers benefit disproportionately from networked water systems, reinforcing spatial inequalities documented by Beard and Mitlin^42^ and Ssemugabo et al.^23^.

The continued dependence on unimproved and surface water in regions such as Bunyoro, Tooro, and Karamoja underscores long-standing structural vulnerabilities in rural and remote areas of Uganda^27^ and mirrors trends observed across other SSA countries^15,43^. Similar findings from Ethiopia, Tanzania, and northern Uganda suggest that geography, water scarcity, and limited infrastructure investment drive reliance on unsafe water sources, particularly where groundwater is inaccessible or highly variable^44^.These patterns support broader regional evidence indicating that rural communities face disproportionate challenges due to hydrological variability, dispersed settlements, and constrained institutional capacity^44,45^. Notably, however, the higher-than-expected proportion of surface water users in Tooro and Ankole diverges from earlier studies reporting greater dependence on groundwater sources in rural Uganda^43,45^. This difference may reflect local environmental pressures, seasonal depletion of shallow wells, or contamination of traditional water sources, factors that merit further investigation.

Refugee settlements experienced the most severe drinking water deficits, with almost no safely managed services and heightened reliance on unimproved and surface water compared with urban and rural populations. This finding is consistent with studies in Uganda’s refugee-hosting districts, which attribute poor water safety to aging infrastructure, inconsistent funding, and high operational demands^46^. It also aligns with recent WASH assessments in humanitarian settings across SSA, which highlight chronic infrastructural constraints, rapid population turnover, and dependence on emergency water provision, such as trucking and temporary boreholes, as key barriers to sustainable safe water access s^17^. Collectively, these results reinforce the widely documented reality that refugee settlements remain among the most underserved and structurally disadvantaged populations in the region.

### Sanitation service levels

The sanitation service results further deepen the picture of widespread inequality across Uganda. The relatively high national proportion of safely managed sanitation (43.6%) contrasts sharply with findings from settlement-level studies in informal urban areas. For instance, Tumwebaze et al.^25^ reported safely managed sanitation as low as 7% in high-density Kampala slums, where overcrowding, shared facilities, and poor waste containment undermine service quality. This contrast suggests that while urban informal settlements perform poorly, many rural and peri-urban households may be achieving safely managed standards through private, improved latrines with adequate containment and disposal mechanisms^26^.

The concentration of safely managed sanitation in regions such as West Nile, Tooro, Ankole, and Acholi is particularly noteworthy, as national assessments, including the most recent census typically classify these regions as having moderate but not exceptional sanitation coverage^9^.This discrepancy implies that service quality within improved rural sanitation systems may be higher than previously recognized, possibly driven by household investment, NGO-led programming, or region-specific sanitation campaigns. These findings highlight the need for further sub-regional analysis to understand the drivers of unexpectedly strong sanitation outcomes in these areas.

The divergence between national-level results and earlier settlement-level studies underscores the substantial variability in sanitation performance across different community types. While slums and other high-density settlements consistently show extremely low safely managed sanitation (often below 10%)^25^, broader nationally representative analyses, including this study, capture a more complex landscape in which some rural and peri-urban households achieve higher service levels. This reinforces the value of national-scale JMP-aligned assessments, which can reveal inequalities that localized case studies might overlook. At the same time, the results remain consistent with regional WASH studies indicating that more than one billion people in Africa still lack safely managed sanitation, despite modest gains in service coverage^18,47^. Persistent structural barriers such as limited faecal sludge management capacity, inadequate sewerage networks, and growing urban populations, continue to hinder progress across many African contexts, including Uganda^15^.

Sanitation conditions in refugee settlements, however, remain critically poor, with only 3.4% of households accessing safely managed sanitation and many relying on inadequate or high-risk options, including open defecation. These results align with regional evaluations of camp-based populations, which consistently document overcrowding, insufficient space for latrine construction, rapid population turnover, and short-term humanitarian planning as major obstacles to achieving sustainable sanitation coverage^17^.The persistence of open defecation, even at low percentages, within refugee settlements underscores the difficulty of eliminating this practice under humanitarian constraints and highlights the need for stronger long-term investment and coordination between humanitarian and government agencies.

### Determinants of household drinking and sanitation service levels

#### Households’ wealth status

A positive association was found between the household wealth status and drinking water and sanitation service levels. Wealthier households are better able to invest in safe water services and improved sanitation facilities, while poorer households often rely on unimproved sources and basic or no sanitation due to financial and infrastructural constraints^37,46,47^. This results mirrors recent research in Kampala, where financial constraints were shown to be a major barrier to adoption of improved sanitation technologies despite awareness of their benefits^25,42^. Other studies have likewise documented high upfront costs for improved latrines, frequent price shocks for piped water, and disproportionate expenditure burdens on low-income households^22,23^. Across SSA, multiple reviews highlight underinvestment, affordability barriers, and unequal cost-sharing arrangements as major obstacles to achieving SDG 6^15,16^. These findings also align with the WHO/UNICEF 2000–2024 global progress assessment, which underscores that safely managed water and sanitation remain heavily skewed toward the richest quintiles in low-income countries^29^. The results from Uganda reaffirm this gradient. Economic stratification determines whether households can move beyond basic or limited services. Low-income and ultra-poor households remain trapped in a cycle of unsafe water and shared sanitation, an inequality pattern consistently observed in both rural and urban SSA contexts^15,16^.

### Place of residence

Urban households exhibited the highest probability of accessing higher water and sanitation service levels, mirroring their greater access to piped infrastructure, regulated services, and utility oversight. This finding is supported by numerous Ugandan studies documenting the concentration of safely managed services in Kampala and secondary cities^21,25,42,48^. Rural households, despite dominating the population, remain heavily reliant on basic and limited services consistent with regional evidence showing slower sanitation adoption, infrastructural deficits, and large reliance on communal sources across SSA’s rural regions^44^. This underscores the urgent need for equitable investment in water and sanitation infrastructure, particularly in rural areas, to reduce disparities and improve overall public health outcomes^15,19^. Refugee settlements exhibited the lowest likelihood of accessing improved services for both water and sanitation. This aligns with humanitarian assessments from Uganda, Ethiopia, and South Sudan which document chronic underfunding, aging borehole systems, and rapid population inflows that overwhelm service capacity^15^. IDP and refugee studies across SSA further confirm that camp environments face severe operational, logistics, and sustainability constraints that impede progress toward safely managed services^17^.These findings reaffirm the geographic and structural disadvantages faced by displaced populations and underscore the need for long-term, durable WASH solutions beyond short-term emergency programming.

#### Sex of household head

The finding that male-headed households had higher likelihood of accessing improved water and sanitation is consistent with a study conducted in rural districts of southern Ghana, where male-headed households were more than twice as likely to have improved waste and sanitation facilities compared to female-headed households^50^. While women shoulder the majority of water collection and household sanitation responsibilities, they often have limited control over financial decision-making, land, and infrastructure investment, which affects their ability to secure high-quality WASH services. Studies in Uganda’s slums have shown that female-headed households tend to experience greater economic vulnerability, more unstable tenancy arrangements, and higher exposure to unsafe or shared facilities^23^. Comparable studies in Kenya, Zimbabwe, and Malawi similarly report that female-headed households face greater access barriers due to income disparities, limited bargaining power with landlords, and lower access to formal water connections^15^. Gendered inequalities in asset ownership and household income also shape sanitation decision-making, with male-headed households more likely to invest in private latrines or pay for desludging services. The JMP 2023 progress reports further highlight that gendered social roles in SSA often place women at the center of WASH burdens while limiting their access to the resources required to upgrade facilities^36^. The results of this study therefore reflect broader structural gender inequities. Despite being disproportionately responsible for water and sanitation management, women in Uganda encounter systemic economic and social barriers that constrain their ability to secure higher service levels. Addressing these inequalities requires integrated gender-responsive WASH programming that improves financial inclusion and asset ownership among women.

#### Education level of household head

The education level of the household head was found to be significantly associated with drinking water sources and sanitation service levels The results show that households whose heads were highly educated had higher probability of accessing improved toilet facilities compared to households whose heads had no education. Generally, education is a resource factor of quality health outcomes in communities in sub-Saharan Africa^38^. Still, people with higher levels of education are often more aware of the importance proper sanitation practices^49^. This study finding is consistent with results from studies conducted in Ethiopia^50^, rural districts of southern Ghana^51^ and in Kampala Slums, Uganda^24,52^. The JMP 2024 global progress report likewise identifies low educational attainment as a structural factor underpinning persistent inequalities in safely managed water and sanitation across SSA^29^. The findings of this study, therefore, reinforce a consistent regional pattern, i.e., education enhances agency and knowledge, enabling households to achieve higher levels of WASH service despite systemic constraints. In contrast, households with low education remain structurally disadvantaged, often reliant on unimproved or shared facilities, echoing wider SSA trends where educational gradients underpin large intra-country WASH disparities^44^. However, the results contradicts with the study findings from poor peri-urban settlements of Abidjan, Côte d’Ivoire^53^. The contradiction between the findings of this study and the study conducted in Abidjan, Ivory Coast, could be attributed to differences in sample size, as the Abidjan study involved only 556 households. This smaller sample size may limit the generalizability of its findings and could contribute to variability in the results compared to the larger, more diverse sample in this study.

#### Region of the household

The pronounced regional disparities observed in the regression analysis, i.e., positive coefficients in South Buganda, North Buganda, Tooro, Ankole, and Bunyoro and negative coefficients in Bukedi, Teso, and Karamoja, mirror longstanding geographic inequalities in Uganda’s WASH landscape. These inequalities are deeply rooted in differences in infrastructure investment, hydrological conditions, population density, and governance capacity. Prior research has consistently shown that central and western Uganda benefit from comparatively stronger piped networks and better-maintained sanitation systems^42^. Similarly, northern and eastern regions, including Karamoja, experience persistent service deficits that could be attributed to arid climates, dispersed settlements, groundwater limitations, and chronic infrastructural underfunding. Recent SSA research supports the same pattern. Rural and semi-arid regions exhibit some of the lowest safely managed drinking water and sanitation rates due to logistical difficulty, high operational costs, and weak institutional presence^15,44^. The WHO/UNICEF JMP 2024 progress assessment also notes that regional disparities within countries are among the most persistent obstacles to universal service, often reflecting political prioritization and capital investment patterns^29^. The extremely low coefficients for Teso, Bukedi, and Karamoja further emphasize areas where Uganda is least likely to reach SDG 6 without targeted interventions. These findings align with humanitarian studies showing that Karamoja and other remote regions face seasonal water shortages, dependence on surface water, and higher open defecation rates—all of which compound structural inequalities^15,42^.

### Household size

This study further found out that the proportion of households with access to improved toilet facilities was higher and significant in households with less than 3 members compared to those with 4 members and above. This result contradicts various studies. For example, studies conducted in Benin^54^, Ethiopia^50^ and Bangladesh^55^ show that larger families had more access to improved toilet facilities than small families. While studies in Benin, Ethiopia, and Bangladesh have found a positive association between large household size and access to improved toilet facilities in their settings, the socioeconomic and housing dynamics in Uganda may lead to an inverse relationship where smaller, often more affluent households, secure improved sanitation more readily than larger families.

### Strength and limitation of the study

A key strength of this study is its use of a nationally representative dataset, which provides the most comprehensive overview to date of drinking water and sanitation service levels across Uganda. Unlike prior research concentrated in Kampala slums or selected districts, these findings offer national generalizability, capturing urban, rural, and refugee settlement contexts. The study also applies the WHO/UNICEF Joint Monitoring Programme (JMP) service ladder framework, marking one of the first attempts to systematically classify Uganda’s water and sanitation services using globally standardized criteria. This enhances comparability across regions and future SDG reporting.

Several limitations should be acknowledged. First, the cross-sectional nature of the dataset restricts causal inference; associations should not be interpreted as definitive causal pathways. Longitudinal data would be needed to assess dynamic changes in service levels over time. Second, the study relies on self-reported household information, including walking time and sanitation type, which may be prone to recall bias or social desiriorability bias. Third, the dataset does not capture seasonal variability, which can affect water availability, quality, and household collection practices in regions experiencing extended dry seasons. Fourth, the JMP classification used here, though globally accepted, may mask micro-level environmental and quality differences within service ladder categories. For example, households classified as having “basic” services may experience different reliability or contamination risks depending on local infrastructure conditions, which could not be fully assessed from the available data. Finally, while refugee settlements were included, data limitations restricted deeper exploration of humanitarian service delivery models, facility age, and operational challenges, factors that uniquely shape WASH outcomes for displaced populations. Future mixed-method or geospatial studies could help address these gaps by integrating system performance data, infrastructure audits, and environmental assessments.

## Conclusion

This study provides the first nationally representative analysis of drinking water and sanitation service levels in Uganda using the WHO/UNICEF JMP service ladder framework, offering a comprehensive assessment of both access and the underlying determinants shaping service inequalities. The findings reveal that despite progress in expanding basic services, safely managed drinking water and sanitation remain limited, with pronounced disparities across socioeconomic groups, regions, and residence types. Both rural and Refugee settlements continue to exhibit low positions on the WHO/UNICEF Joint Monitoring Programme (JMP) service ladders. Education, wealth, gender of household head, and geographic location emerge as the strongest and most consistent predictors of service levels, underscoring the structural nature of WASH inequality in the country. Regional differences, particularly those disadvantaging Bukedi, Teso, Karamoja, and refugee settlements, highlight the need for targeted investments and policy interventions that prioritize underserved populations. The persistent disadvantages faced by low-income and female-headed households further demonstrate that progress toward universal WASH access cannot be achieved without addressing broader socioeconomic inequities. By integrating the JMP framework with robust statistical modeling, this study fills a critical evidence gap and provides actionable insights for policymakers, practitioners, and researchers working to advance SDG 6 in Uganda. Strengthening human capital, reducing financial barriers, improving infrastructure in remote regions, and embedding equity-focused approaches within national WASH programming will be essential for accelerating progress. Continued investment in data systems, monitoring, and context-specific research will further support the development of resilient, inclusive, and sustainable WASH services for all Ugandans. Future research should build on this work through longitudinal analyses, geospatial mapping, and mixed-methods studies that uncover how education, wealth, gender, and regional dynamics shape service inequality, ultimately supporting more effective and context-responsive strategies for achieving universal WASH access.

## Data Availability

The data used in this study can be obtained by sending a request via the DHS Program website and upon approval data can be obtained from https://dhsprogram.com/data/dataset/Uganda_MIS_2018.cfm?flag=1.

## Abbreviations

CAPI: Computer Assisted Personal Interviewing
DHS: Demographic and Health survey
EA: Enumeration Area
JMP: Joint Monitoring Programme
ICF: Inner-City Fund
MoH: Ministry of Health
MWE: Ministry of Water and Environment
NMCD: National Population and Housing Census
SDG: Sustainable Development Goal
SSA: Sub Saharan Africa
UBOS: Uganda Bureau of Statistics
UMIS: Uganda Malaria Indicator Survey
WHO: World Health Organization

## References

[1] WHO. *Water, Sanitation, hygiene and health: A primer for health professionals*. *World Heal Organ* (2021).

[2] Jeuland, M. A., Fuente, D. E., Ozdemir, S., Allaire, M. C. & Whittington, D. The Long-Term dynamics of mortality benefits from improved water and sanitation in less developed countries. *PLoS One* **8**, e74804, 1–16 (2013). 10.1371/journal.pone.0074804 PMC3792953 24116011

[3] WHO & UNICEF. Progress on Household Drinking Water, Sanitation and Hygiene (Jmp). (2023).

[4] Stringer, L. C. et al. Climate change impacts on water security in global drylands. *One Earth*. **4**, 851–864 (2021).

[5] McCaw-Binns, A. & Hussein, J. The millennium development goals. *Matern Perinat. Heal Dev. Ctries.* 10–24. 10.1079/9781845937454.0010 (2012).

[6] Akrash. *Progress on drinking water, sanitation and hygiene in Africa 2000–2020 5 years into the SDGs*. *United Nations Child. Fund World Heal Organ* (2020).

[7] WHO & UNICEF. Progress on household drinking water, sanitation and hygiene 2000–2020: five years into the SDGs. *Jt Water Supply Sanit. Monit. Program* (2021).

[8] Biswas, A. K. & Tortajada, C. Providing clean and safe water to all: A global perspective. *Univer-cities Reshaping Strateg Meet Radic Chang. Pandemics Inequal. - Revisiting Soc. Compact*. **4**, 117–138 (2021).

[9] Uganda Bureau of Statistics (UBOS). National Population And Housing Census 2024. at https://www.ubos.org/wp-content/uploads/2024/12/National-Population-and-Housing-Census-2024-Final-Report-Volume-1-Main.pdf (2024).

[10] Manetu, W. M. & Karanja, A. M. Waterborne disease risk factors and intervention practices: A review. *OALib* **08**, 1–11 (2021).

[11] Mutono, N. et al. The nexus between improved water supply and water-borne diseases in urban areas in africa: a scoping review. *AAS Open. Res.* **4**, 27 (2021). 34368620 10.12688/aasopenres.13225.1 PMC8311817

[12] Chaitkin, M. et al. Estimating the cost of achieving basic water, sanitation, hygiene, and waste management services in public health-care facilities in the 46 UN designated least-developed countries: a modelling study. *Lancet Glob Heal*. **10**, e840–e849 (2022). 10.1016/S2214-109X(22)00099-7 PMC9090898 35397226

[13] Mead, S. *State of the world’s*. *United Nations Child. Fund World Heal Organ. (WHO)*, (2020).

[14] UNICEF & School Water Sanitation, and hygiene: A systematic review of an effect on Health, Attendance, Regularity, and educational achievements. *Sch. J.* 1–21. 10.3126/scholars.v5i1.55744 (2023).

[15] Hlongwa, N., Nkomo, S. L. & Desai, S. A. Barriers to water, sanitation, and hygiene in Sub-Saharan africa: a mini review. *J. Water Sanit. Hyg. Dev.* **14**, 497–510 (2024).

[16] Agbo, C., Jeffrey, P. & Sule, M. N. Evaluation of failings in urban water supply and sanitation systems in Sub-Saharan africa: a systematic review to inform future planning. *J. Water Sanit. Hyg. Dev.* **15**, 148–165 (2025).

[17] Hamza, A. et al. Emergency Water, Sanitation, and Hygiene (wash) Management in the Internally Displaced Persons Camps in Sub-Saharan Africa : A Systematic Review. Nigerian Journal of Water, Sanitation, and Hygiene Development, **1**, 227–244 (2025).

[18] Dinka, M. O. & Nyika, J. SDG 6 progress analyses in sub-Saharan Africa from 2015–2020: the need for urgent action. *Discov Water* **4**, 4–39 (2024).

[19] Okesanya, O. J. et al. Water, sanitation, and hygiene (WASH) practices in africa: exploring the effects on public health and sustainable development plans. *Trop Med. Health* **52**, 52–68 (2024). 10.1186/s41182-024-00614-3 PMC11463047 39385262

[20] Hirai, M., Roess, A., Huang, C. & Graham, J. Exploring geographic distributions of high-risk water, sanitation, and hygiene practices and their association with child diarrhea in Uganda. *Glob Health Action* **9**, 1–9 (2016). 10.3402/gha.v9.32833 PMC5084380 27790971

[21] Sempewo, J. I. & Kayaga, S. Determinants of transitions in drinking water service systems in developing economies: A case study of Uganda. *Water Supply*. **23**, 3532–3551 (2023).

[22] Ssemugabo, C. et al. Knowledge and practices of households on safe water chain maintenance in a slum community in Kampala City, Uganda. *Environ. Health Prev. Med.* **24**, 1–9 (2019). 31200642 10.1186/s12199-019-0799-3 PMC6570909

[23] Ssemugabo, C., Halage, A. A., Namata, C., Musoke, D. & Ssempebwa, J. C. A socio-ecological perspective of the facilitators and barriers to uptake of water, sanitation and hygiene interventions in a slum setting in kampala, uganda: A qualitative study. *J. Water Sanit. Hyg. Dev.* **10**, 227–237 (2020).

[24] Dickson-Gomez, J. et al. Water, Sanitation, and hygiene challenges in informal settlements in Kampala, uganda: A qualitative study. *Int J. Environ. Res. Public. Health* **20**, 61–81(2023). 10.3390/ijerph20126181 PMC10298274 37372767

[25] Tumwebaze, I. K. et al. Access to and factors influencing drinking water and sanitation service levels in informal settlements: evidence from Kampala, Uganda. *Habitat Int.* **136**, 102829 (2023).

[26] Kayiwa, D. et al. Assessment of water, sanitation and hygiene service availability in healthcare facilities in the greater Kampala metropolitan area, Uganda. *BMC Public. Health*. **20**, 1–11 (2020). 33228619 10.1186/s12889-020-09895-9 PMC7682765

[27] Colding-Jørgensen, J. T., Muheki, E., Baayenda, G. & Harding-Esch, E. Assessing Water, sanitation and hygiene access and use in Nabilatuk District, uganda: A Cross-Sectional study of different data collection methods. *Hygiene* **3**, 65–84 (2023).

[28] Bosco Isunju, J. et al. Assessment of the status of Water, Sanitation and Hygiene in Healthcare Facilities in the Greater Kampala Metropolitan Area Final report. at https://washmatters.wateraid.org/sites/g/files/jkxoof256/files/2021-11/WaterAid Uganda Final WASH in HCFs Assesment Final Report July 2019....FIN %281%29.pdf (2019).

[29] WHO & UNICEF. Progress on Household Drinking Water, Sanitation and Hygiene 2000–2024. UNICEF J. at https://www.eea.europa.eu/publications/industrial-waste-water-treatment-pressures%0Ahttp://files/558/Rapport (2024). EEA Industrial waste water treatment – pressures on Europe’s environment.pdf&gt

[30] Climate Risk Profile-Uganda. Climate risk country profile: Uganda. *World Bank. Gr* 32 (2020).

[31] Uganda Bureau of Statistics, (UBOS). National population and housing census 2014—main report, Uganda Bureau of Statistics (UBOS): Kampala, Uganda, 2016. (2016).

[32] Office of the Prime Minister (OPM) and United Nations Higher Commissioner for Refugees (UNHCR). Uganda- Population Dashboard: Overview of Refugees and Asylum-seekers in Uganda. 0–1. (2023).

[33] Uganda Bureau of Statistics. Statistical Abstract. *Uganda Bur. Stat. Stat.* 1–336 (2022). (2022).

[34] Uganda National Malaria Control Division (NMCD). Uganda bureau of statistics (UBOS), and ICF. Uganda malaria indicator survey 2018-19. *Kampala Uganda* **52** (2020).

[35] Semakula, H. M. et al. Determinants of malaria infections among children in refugee settlements in Uganda during 2018–2019. *Infect. Dis. Poverty*. **3**, 1–12 (2023). 10.1186/s40249-023-01090-3 PMC10084630 37032366

[36] Unicef & World Health Organisation. Progress on household drinking water, sanitation and hygiene 2000–2022: special focus on gender. at https://www.who.int/publications/b/69664 (2023).

[37] Gashaneh, D., Id, B. & Andualem, Z. Limited access to improved drinking water, unimproved drinking water, and toilet facilities among households in Ethiopia : Spatial and mixed effect analysis. 1–20 (2022). 10.1371/journal.pone.0266555 10.1371/journal.pone.0266555 PMC8975151 35363836

[38] Agbadi, P., Darkwah, E. & Kenney, P. L. A Multilevel Analysis of Regressors of Access to Improved Drinking Water and Sanitation Facilities in Ghana. *J. Environ. Public Health* **2019**, (2019). 10.1155/2019/3983869 PMC6589203 31275403

[39] Semakula, H. M., Song, G., Zhang, S. & Achuu, S. P. Potential of household environmental resources and practices in eliminating residual malaria transmission: A case study of tanzania, burundi, Malawi and Liberia. *Afr Health Sci* **15**, 819–827 (2015). 10.4314/ahs.v15i3.16 PMC4765464 26957970

[40] Nygren, B. L. et al. The relationship between distance to water source and moderate-to-severe diarrhea in the global enterics multi-center study in Kenya, 2008–2011. *Am. J. Trop. Med. Hyg.* **94**, 1143–1149 (2016). 26928833 10.4269/ajtmh.15-0393 PMC4856616

[41] ICF. Demographic and Health Surveys Standard Recode Manual for DHS 7. The Demographic and Health Surveys Program. Rockville, Maryland, U.S.A.: ICF. 1–204 at https://dhsprogram.com/pubs/pdf/DHSG4/Recode7_DHS_10Sep2018_DHSG4.pdf (2018).

[42] Beard, V. A. & Mitlin, D. Water access in global South cities: the challenges of intermittency and affordability. *World Dev.* **147**, 105625 (2021).

[43] Demoze, L. et al. Determinants and geographic distribution of unimproved sanitation facilities in sub-Saharan Africa, Spatial and multilevel analysis using demographic and health survey (DHS) data. *BMC Public. Health* **25**, 2–16 (2025). 10.1186/s12889-025-24184-z PMC12366091 40830938

[44] Gedamu, G. et al. Barriers to the sustainability of rural water schemes in Sub-Saharan African countries: a systematic review. *J. Water Sanit. Hyg. Dev.* **15**, 427–442 (2025).

[45] Nyika, J. & Dinka, M. O. *Water Challenges in Rural and Urban Sub-Saharan Africa and their Management*. 10.1007/978-3-031-26271-5_5 (2023).

[46] Pearce, J. et al. Water supply for refugees and their host communities in protracted situations: costs and financing options for sustaining services in Ethiopia and Uganda. *J. Water Sanit. Hyg. Dev.* **14**, 1260–1268 (2024).

[47] Bishoge, O. K. Challenges facing sustainable water supply, sanitation and hygiene achievement in urban areas in sub-Saharan Africa. *Local. Environ.* **26**, 893–907 (2021).

[48] Tsimpo, C. & Wodon, Q. Water and sanitation in Uganda. *Water Sanit. Uganda*. 10.1596/978-1-4648-0711-4 (2018).

[49] Tseole, N. P., Mindu, T., Kalinda, C. & Chimbari, M. J. Barriers and facilitators to Water, sanitation and hygiene (WaSH) practices in Southern africa: A scoping review. *PLoS One*. **17**, 1–17 (2022). 10.1371/journal.pone.0271726 PMC9345477 35917339

[50] Andualem, Z. et al. Households access to improved drinking water sources and toilet facilities in ethiopia: A multilevel analysis based on 2016 Ethiopian demographic and health survey. *BMJ Open* **11**, e042071, 1–9 (2021). 10.1136/bmjopen-2020-042071 PMC7978246 33737423

[51] Akpakli, D. E., Manyeh, A. K., Akpakli, J. K., Kukula, V. & Gyapong, M. Determinants of access to improved sanitation facilities in rural districts of Southern ghana: evidence from Dodowa health and demographic surveillance site. *BMC Res. Notes*. **11**, 1–7 (2018). 30005694 10.1186/s13104-018-3572-6 PMC6045853

[52] Kwiringira, J., Atekyereza, P., Niwagaba, C. & Günther, I. Gender variations in access, choice to use and cleaning of shared latrines; experiences from Kampala Slums, Uganda. *BMC Public. Health*. **14**, 1–11 (2014). 25407788 10.1186/1471-2458-14-1180 PMC4247598

[53] Dongo, K., Templeton, M. R., Zinsstag, J. & Bonfoh, B. Barriers to access improved water and sanitation in poor peri-urban settlements of Abidjan, Côte d’ivoire. PLoS ONE **13**(8): e0202928.1–13 (2018). 10.1371/journal.pone.0202928 PMC6112649 30153297

[54] Gaffan, N. et al. Household access to basic drinking water, sanitation and hygiene facilities: secondary analysis of data from the demographic and health survey V, 2017–2018. *BMC Public. Health*. **22**, 1–17 (2022). 35836162 10.1186/s12889-022-13665-0 PMC9284778

[55] Sabbir Ahmed, M. I., Islam, I., Chandra Das, M., Khan, M. & Md Yunus, F. A. I. Mapping and situation analysis of basic WASH facilities at households in bangladesh: evidence from a nationally representative survey. (2021). 10.1371/journal.pone.0259635 10.1371/journal.pone.0259635 PMC8568162 34735535

